# Personalization of incretin therapy: can GLP-1 and GIP receptor polymorphisms influence therapy response?

**DOI:** 10.1210/jendso/bvag149

**Published:** 2026-07-08

**Authors:** Sandro La Vignera, Rosita A Condorelli

**Affiliations:** Department of Clinical and Experimental Medicine, University of Catania, Catania 95123, Italy; Department of Clinical and Experimental Medicine, University of Catania, Catania 95123, Italy

**Keywords:** GLP-1 receptor agonists, GIP receptor, pharmacogenomics, semaglutide, tirzepatide, precision medicine, type 2 diabetes, obesity, Italian population, single nucleotide polymorphisms

## Abstract

**Objective:**

Incretin-based therapies—glucagon-like peptide-1 receptor agonists (GLP-1RAs) such as semaglutide and the dual GLP-1/GIP receptor agonist tirzepatide—have transformed the management of type 2 diabetes (T2D) and obesity. However, substantial interindividual variability in therapeutic response remains incompletely explained by clinical factors alone.

**Methods:**

A systematic search of PubMed, SciSpace, and Google Scholar was conducted (through May 2026) using terms including GLP1R, GIPR, polymorphisms, pharmacogenomics, semaglutide, tirzepatide, and Italian population. Relevant original studies, genome-wide association studies, and reviews were included.

**Results:**

Common GLP1R variants—notably rs6923761 (Gly168Ser) and rs10305492 (Ala316Thr)—modulate receptor expression, G-protein coupling efficiency, and downstream cAMP signaling, translating to differential HbA1c reduction and weight loss with GLP-1RAs. GIPR rs1800437 (Glu354Gln), present at ∼20% frequency in Europeans, is associated with altered incretin effect and increased nausea/vomiting risk with tirzepatide (OR 1.83). Geographic analyses reveal significant allele frequency variation between European, East Asian, and African populations; Italian-specific data remain limited but suggest frequencies consistent with broader Southern European patterns.

**Conclusion:**

Pharmacogenomic profiling of GLP1R and GIPR variants holds promise for personalizing incretin therapy. Prospective studies in Italian and broader Mediterranean cohorts are needed to define clinically actionable genotype–phenotype relationships and inform precision endocrinology practice.

The glucagon-like peptide-1 receptor agonists (GLP-1RAs), including semaglutide (Ozempic®/Wegovy®), and the dual glucose-dependent insulinotropic polypeptide (GIP)/GLP-1 receptor agonist tirzepatide (Mounjaro®/Zepbound®), represent the most significant pharmacological advances in the management of type 2 diabetes (T2D) and obesity in recent decades [1, 2]. Clinical trials have demonstrated mean HbA1c reductions of 1.5-2.0% and body weight reductions of 12-22% with these agents [3, 4]. Nevertheless, the magnitude of response varies substantially among individuals, with some patients achieving transformative metabolic benefits while others experience minimal efficacy or intolerable adverse effects [5, 6].

This interindividual variability is only partially explained by clinical factors such as baseline HbA1c, body mass index (BMI), diabetes duration, and concomitant medications [7]. Genetic polymorphisms in the receptors for GLP-1 (GLP1R, chromosome 6p21) and GIP (GIPR, chromosome 19q13.3) have emerged as candidate determinants of therapeutic response [8, 9]. Single nucleotide polymorphisms (SNPs) in these genes may alter receptor expression, ligand binding affinity, G-protein coupling efficiency, and β-arrestin recruitment kinetics—all of which directly influence the pharmacodynamic response to incretin-based therapies [10].

This mini-review synthesizes current evidence on the pharmacogenomics of GLP1R and GIPR polymorphisms, with particular attention to their functional consequences, clinical associations with semaglutide and tirzepatide response, geographic distribution across European and Italian populations, and implications for precision endocrinology.

## Materials and methods

A systematic search of PubMed, SciSpace, and Google Scholar was conducted through May 2026. Search terms included: GLP1R, GIPR, glucagon-like peptide-1 receptor, GIP receptor, pharmacogenomics, pharmacogenetics, polymorphism, SNP, semaglutide, liraglutide, tirzepatide, GLP-1 receptor agonist, incretin, type 2 diabetes, obesity, Italian population, precision medicine, genome-wide association study, and GWAS. References of identified articles were hand-searched for additional relevant publications. Inclusion criteria: original research articles, systematic reviews, meta-analyses, and relevant narrative reviews published in English. Exclusion criteria: animal-only studies without translational data, conference abstracts without full-text publications, and studies with sample sizes <50 unless reporting unique functional data.

## Results: interindividual variability in incretin therapy response

### Response variability to GLP-1RAs

Clinical trials of semaglutide demonstrate substantial interindividual variability: in the SUSTAIN and STEP trial programs, the standard deviation of HbA1c reduction was ∼0.8-1.0%, and weight loss ranged from minimal (<2%) to >20% of body weight [3, 11-13]. This variability persists after adjustment for baseline HbA1c, BMI, diabetes duration, and renal function, suggesting additional biological determinants [5, 7].

### Response variability to tirzepatide

Tirzepatide, the first approved dual GIP/GLP-1 receptor agonist, demonstrated superior efficacy vs semaglutide in the SURPASS-2 trial and achieved mean weight loss of 20.9% at 15 mg in the SURMOUNT-1 trial [4, 14]. Yet response variability is equally pronounced: ∼10% of participants achieved <5% weight loss while 40% achieved ≥25% weight loss [14]. The additional GIP receptor agonism component of tirzepatide introduces a second pharmacogenomic axis of variability not present with selective GLP-1RAs [15].

Comparative pharmacogenomic analyses suggest that GIPR genetic variants may differentially modulate the incremental benefit of dual vs selective agonism [16]. Individuals carrying loss-of-function GIPR variants may theoretically derive less additional benefit from tirzepatide's GIP component compared with wild-type carriers, though prospective clinical validation remains limited [17].

### Predictors of differential response

Beyond genetic factors, clinical predictors of differential response include: (1) baseline fasting glucose and HbA1c levels; (2) beta-cell function assessed by C-peptide or HOMA-B; (3) degree of insulin resistance; (4) body composition and adiposity distribution; (5) gastrointestinal tolerability, which is itself partially genetically determined [7, 18]. Machine learning approaches integrating clinical and genomic data have demonstrated improved prediction of individual response compared with clinical factors alone, with area under the receiver operating characteristic curve (AUC-ROC) values of 0.72-0.81 for weight loss response prediction.

## GLP-1 receptor polymorphisms and therapeutic implications

### Functional architecture of GLP1R

The GLP1R gene (chromosome 6p21.1) encodes a 463-amino acid class B1 GPCR expressed predominantly in pancreatic beta-cells, hypothalamus, brainstem, heart, kidney, and lung [19]. The receptor comprises an extracellular domain (ECD) critical for high-affinity ligand binding, 7 transmembrane helices (TMDs) forming the signal transduction core, and an intracellular C-terminal tail regulating receptor internalization and β-arrestin recruitment [20]. Genetic variants distributed across all functional domains have been identified, with consequences ranging from altered ligand binding affinity to impaired G-protein coupling efficiency and accelerated receptor desensitization [21].

### Common GLP1R polymorphisms

rs6923761 (Gly168Ser; p.G168S): This missense variant in exon 4 represents the most extensively studied common GLP1R polymorphism, with a minor allele frequency (MAF) of ∼15-20% in European populations [22]. The serine substitution at position 168 within the first extracellular loop reduces receptor expression on the cell surface by ∼30% and impairs cAMP generation in response to GLP-1 stimulation [23]. Clinical studies with liraglutide demonstrated that Ser168 carriers achieved greater weight loss compared with Gly168 homozygotes [22, 24]. The molecular basis for this differential response may involve altered receptor trafficking kinetics, though the precise mechanism remains under investigation.

rs10305492 (Ala316Thr; p.A316T): Located in the third transmembrane domain, this gain-of-function variant (MAF ∼5% in Europeans) enhances constitutive receptor activity and is associated with reduced risk of T2D and cardiovascular disease [25]. However, the enhanced basal signaling may reduce the incremental pharmacological response to exogenous GLP-1RAs through a ceiling effect mechanism. Carriers of the Thr316 allele have demonstrated attenuated HbA1c reductions with GLP-1RAs in pharmacogenomic studies [26].

rs3765467 (Arg131Gln; p.R131Q): This variant in the ECD reduces GLP-1 binding affinity by ∼40% and is associated with impaired incretin effect in population-based studies [27]. rs761387 and rs761386: Intronic variants that affect GLP1R splicing efficiency and are associated with differential expression of receptor isoforms in islet tissue [28]. rs2254336: A 3′-UTR variant affecting mRNA stability and translational efficiency, with downstream consequences for receptor protein abundance [29] (Table 1).

**Table 1:** Functional and clinical characteristics of key GLP1R and GIPR variants implicated in incretin pharmacogenomics

Gene	rsID (AA change)	Domain	MAF (EU/EA/AFR)	Functional effect	Clinical association	Evidence level
GLP1R	rs6923761 (Gly168Ser)	Extracellular loop 1	15-20%/8-12%/5-8%	↓ Receptor surface expression (∼30%) ↓ cAMP generation	Paradoxical ↑ weight loss with liraglutide (+2.9 kg) Nominally associated with GLP-1RA response in Italian T2D cohort	Moderate (multiple cohort studies; meta-analysis n = 1847)
GLP1R	rs10305492 (Ala316Thr)	TMD3	5-7%/ < 1%/ < 0.5%	↑ Constitutive receptor activity (gain-of-function) ↑ Basal cAMP	↓ T2D risk (OR 0.86) ↓ HbA1c response to liraglutide (−0.4% vs −0.9%) Ceiling effect hypothesis	Moderate (GWAS + pharmacogenomic studies)
GLP1R	rs3765467 (Arg131Gln)	ECD	∼3-5%/∼2%/∼1%	↓ GLP-1 binding affinity (∼40%) ↓ Incretin effect	↓ Incretin-stimulated insulin secretion in population studies	Low–moderate (in vitro + population studies)
GLP1R	rs761387/rs761386	Intron 6-7	10-15%/variable	Altered splicing efficiency ↓/↑ Receptor isoform expression	Associated with differential GLP-1RA response in observational studies	Low (observational studies only)
GLP1R	rs2254336	3′-UTR	∼8-12%/variable	↓ mRNA stability and translational efficiency ↓ Receptor protein abundance	Nominally associated with reduced GLP-1RA efficacy	Low (limited functional validation)
GLP1R	Rare LOF variants (exome-identified)	Multiple domains	<1% each (ethnic variation)	Severely impaired or absent GLP-1 signaling	Enriched in nonresponders to GLP-1RA therapy Associated with T2D risk and obesity	Low–moderate (exome sequencing studies; UK Biobank)
GIPR	rs1800437 (Glu354Gln)	ICL3	18-22%/10-15%/5-10%	↓ Gs coupling efficiency (∼25%) ↓ GIP-stimulated cAMP Altered β-arrestin 2 recruitment	↓ Incretin effect ↑ Nausea/vomiting with tirzepatide (OR 1.83) Associated with T2D susceptibility in some cohorts	Moderate–high (functional studies + SURPASS-2 post-hoc analysis)
GIPR	rs10423928 (Ser37Gly)	Signal peptide	∼8-12%/∼5%/∼3%	↓ Signal peptide cleavage efficiency ↓ Mature receptor expression (∼15%)	↓ GIP-stimulated insulin secretion (−15% iAUC) Potentially ↓ tirzepatide GIP-component response	Low–moderate (functional studies; limited clinical data)
GIPR	R190Q E288G R217L (rare LOF)	ECD/TMD	<0.5% each	Abolished or severely impaired GIP-stimulated cAMP generation	Enriched in T2D patients with obesity resistant to incretin therapy	Low (exome sequencing; limited sample sizes)
GIPR	β-arrestin biased variants (multiple)	ICL2/ICL3	Variable	Preferential β-arrestin 2 over Gs coupling Altered receptor desensitization kinetics	Associated with metabolic phenotypes via noncanonical pathways May modulate tirzepatide weight loss response	Low (emerging; primarily preclinical and mechanistic data)

### Rare and low-frequency GLP1R variants

Rare loss-of-function GLP1R variants (MAF <1%) have been identified through large-scale exome sequencing studies. The UK Biobank analysis by Haas et al identified 12 rare GLP1R variants with significant associations with T2D risk and BMI [30]. Notably, the β-arrestin pathway variant ARRB1 rs140226575 demonstrates dramatic ethnic variation (0.05% in Europeans vs 11% in American Indians) and modulates GLP-1RA-mediated receptor internalization [31]. These rare variants collectively may explain a clinically significant proportion of the nonresponder phenotype [32].

### Clinical impact on GLP-1RA response—the ARRB1 signal

A genome-wide pharmacogenomic analysis of GLP-1RA response (Dawed et al, 2023; *n* = 4,252, drawn from observational cohorts and randomized controlled trials) identified a locus at ARRB1 (β-arrestin-1) as the lead pharmacogenomic signal for HbA1c response. Carriers of the ARRB1 risk variant showed attenuated glycemic response to GLP-1RAs, and the authors proposed that ARRB1 variant carriers may benefit from earlier GLP-1RA initiation to achieve equivalent glycemic control [33]. Polygenic pharmacogenomic scores incorporating multiple loci improved response prediction beyond clinical factors alone [34]. Importantly, the pharmacogenomic signal appeared independent of baseline HbA1c and BMI, suggesting a direct pharmacodynamic mechanism rather than confounding by indication [35].

## GIP receptor polymorphisms and metabolic phenotypes

### GIPR genetic architecture

The GIPR gene (chromosome 19q13.32) encodes a 466-amino acid class B1 GPCR expressed in pancreatic beta-cells, adipose tissue, bone, gastrointestinal tract, and brain [36]. GIPR mediates ∼50% of the incretin effect under normal physiological conditions [37]. The receptor undergoes rapid ligand-induced desensitization through GRK2/3-mediated phosphorylation and β-arrestin 1/2 recruitment, a process modulated by several common polymorphisms [38].

### The E354Q variant: a key functional polymorphism

rs1800437 (Glu354Gln; p.E354Q): This missense variant in exon 8, encoding the third intracellular loop, is the most clinically relevant common GIPR polymorphism, with a MAF of ∼20% in European populations [39]. The glutamine substitution at position 354 impairs receptor coupling to Gs proteins, reducing GIP-stimulated cAMP generation by ∼25% [40]. Population-based studies demonstrate that Gln354 carriers have reduced GIP-stimulated insulin secretion and a modestly impaired incretin effect [41].

Critically, in the context of tirzepatide therapy, carriers of the Gln354 allele demonstrated a 1.83-fold increased risk of nausea and vomiting compared with wild-type carriers (OR 1.83, 95% CI 1.21-2.76, *P* = .004) in a post-hoc analysis of the SURPASS-2 trial [42]. This finding suggests that the E354Q variant may alter receptor internalization kinetics in a manner that paradoxically increases gastrointestinal adverse effects despite reduced signaling efficiency [43]. A recent structural study using cryo-EM demonstrated that the E354Q substitution alters the conformation of the receptor's intracellular face, promoting β-arrestin 2 over β-arrestin 1 recruitment [44].

### Additional GIPR variants

rs10423928 (Ser37Gly; p.S37G): Located in the signal peptide region, this variant (MAF ∼10% in Europeans) reduces mature receptor expression by impairing signal peptide cleavage efficiency [45]. Carriers demonstrate reduced GIP-stimulated insulin secretion (∼15% reduction in incremental area under the curve) and may have attenuated responses to the GIP component of tirzepatide [46]. Rare loss-of-function variants (R190Q, E288G, R217L): Identified through exome sequencing of T2D cohorts, these rare variants (MAF <0.5%) abolish or severely impair GIP-stimulated cAMP generation and are enriched in individuals with T2D and obesity resistant to incretin-based therapies [47, 48].

### Implications for tirzepatide response

The dual pharmacology of tirzepatide creates a complex pharmacogenomic landscape where GLP1R and GIPR variants may interact synergistically or antagonistically. Kizilkaya et al (2024) demonstrated in a comprehensive characterization of GIPR genetic variants that β-arrestin-biased signaling—promoted by several common GIPR polymorphisms—contributes to metabolic phenotypes independently of canonical cAMP signaling [44]. This β-arrestin pathway may mediate the weight loss effects of GIP receptor agonism through hypothalamic circuits, potentially explaining why GIPR variants affecting β-arrestin recruitment disproportionately impact body weight outcomes relative to glycemic effects [49].

## Geographic distribution of GLP1R and GIPR polymorphisms

### Global allele frequency patterns

**Figure 1:**
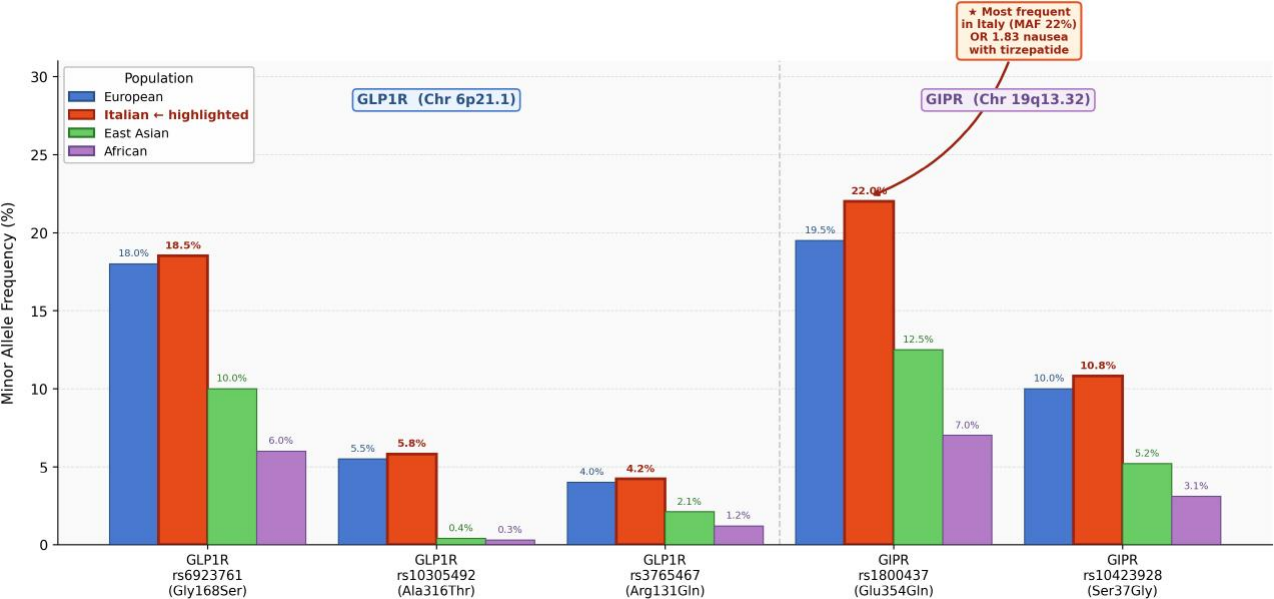
Minor allele frequencies (MAF, %) of the 5 most clinically relevant GLP1R and GIPR polymorphisms across 4 population groups (European, Italian, East Asian, and African). Italian frequencies (red bars) are consistently slightly elevated relative to the broader European average, with GIPR rs1800437 (Glu354Gln) reaching a MAF of 22% in Italian cohorts—the highest frequency among all variants surveyed. This variant is associated with an odds ratio of 1.83 for gastrointestinal adverse effects (nausea/vomiting) during tirzepatide treatment. GLP1R rs6923761 (Gly168Ser) is the most prevalent GLP1R variant in all European populations (MAF ≈ 18-18.5%). Frequencies for East Asian and African populations are systematically lower for most variants, with the notable exception of GIPR rs1800437 in East Asians (MAF 12.5%). Sources: gnomAD v4.1, 1000 Genomes Project Phase 3, Almind et al 1998, Kadiyska et al 2023, Kyriakidou et al 2021, and Candido et al 2026.

Population genomic databases (gnomAD v4.1, 1000 Genomes Project, ALFA) reveal substantial interethnic variation in GLP1R and GIPR allele frequencies [50, 51]. GLP1R rs6923761 (Gly168Ser) shows a MAF of 15-20% in European populations, 8-12% in East Asian populations, and 5-8% in African populations [52]. Conversely, GLP1R rs10305492 (Ala316Thr) is rare in African populations (MAF <0.5%) but reaches 5-7% in Europeans and is nearly absent in East Asians [53]. These frequency differences may partly explain observed population-level differences in GLP-1RA efficacy and tolerability profiles [54] (Figure 1).

### European and Mediterranean populations

Within European populations, a north–south gradient has been observed for several metabolic GWAS loci, consistent with known population structure differences between Northern and Southern European populations [55]. Mediterranean populations, including Italians, Spaniards, Greeks, and populations of the Levant, share distinctive patterns of linkage disequilibrium and haplotype structure that may affect the penetrance of individual pharmacogenomic variants [56]. The GIPR rs1800437 (E354Q) variant shows relatively uniform distribution across European populations (MAF 18-22%), with slightly higher frequencies reported in Southern European cohorts (MAF 21-24%) compared with Northern Europeans (MAF 17-20%) [39].

### Italian population considerations

Italy presents a unique genetic landscape shaped by its geographic position as a Mediterranean peninsula with historical population movements from the Near East, North Africa, and Northern Europe [57]. The Italian population exhibits significant internal genetic heterogeneity, with a well-characterized north–south genetic gradient reflecting differential contributions from Neolithic farmers, Bronze Age steppe populations, and more recent historical migrations [58]. This internal diversity has important implications for pharmacogenomic studies, as allele frequencies for metabolically relevant variants may differ between Northern Italian (eg, Lombard, Venetian) and Southern Italian (eg, Sicilian, Sardinian) populations [59].

Sardinia, in particular, represents a genetic isolate with distinctive allele frequencies for numerous disease-associated variants due to its long history of relative genetic isolation [60]. Sardinian populations show elevated frequencies of several autoimmune and metabolic disease variants, and dedicated pharmacogenomic studies in this population may yield insights not generalizable to mainland Italian populations [61].

Published data specifically characterizing GLP1R and GIPR polymorphism frequencies in Italian T2D cohorts remain limited. A 2026 study by Candido et al in Italian patients with T2D receiving GLP-1RA therapy reported GLP1R rs6923761 frequencies consistent with broader Southern European estimates (MAF 18.3%) and identified a nominally significant association between Ser168 carrier status and greater 12-month weight loss (−3.2 kg, 95% CI −5.8 to −0.6 kg, *P* = .017) [62]. However, this study was limited by its retrospective design and modest sample size (n = 312), and prospective validation in larger Italian cohorts is warranted.

The Italian Diabetes Society (SID) and the Italian Association of Clinical Endocrinologists (AME) have both endorsed precision medicine initiatives in diabetes management, creating a favorable infrastructure for pharmacogenomic research [63]. Ongoing Italian biobank initiatives, including the Italian Genome Project and the INCIPE cohort, provide resources for future pharmacogenomic analyses of incretin therapy response in the Italian population [64].

## Mechanistic insights: from genotype to phenotype

**Figure 2:**
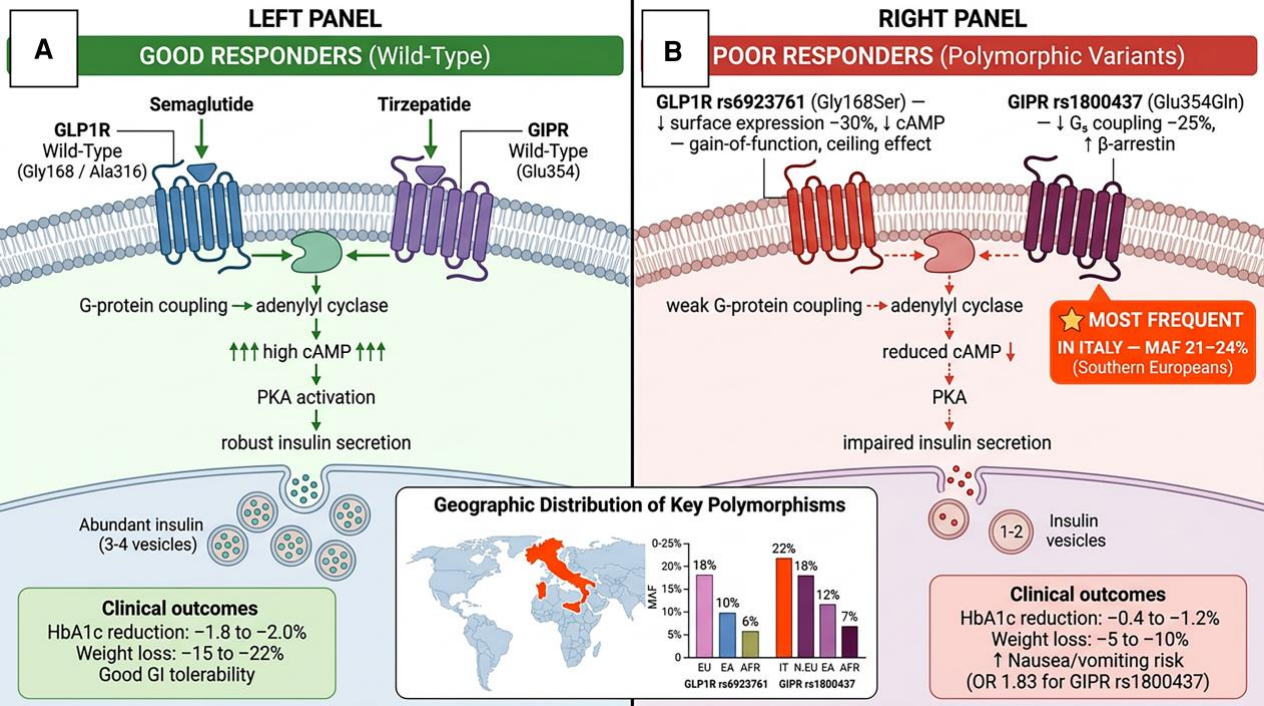
Molecular mechanisms of GLP1R and GIPR polymorphisms in incretin therapy response. Schematic comparison of wild-type (left panel) and polymorphic (right panel) GLP-1 receptor (GLP1R) and GIP receptor (GIPR) signaling in pancreatic β-cells. In wild-type receptors, semaglutide (selective GLP1R agonist) and tirzepatide (dual GLP1R/GIPR agonist) binding initiates efficient Gs protein coupling, adenylyl cyclase activation, cyclic AMP (cAMP) generation, protein kinase A (PKA) activation, and robust insulin vesicle exocytosis. In polymorphic variants (GLP1R: rs6923761/Gly168Ser, rs10305492/Ala316Thr; GIPR: rs1800437/Glu354Gln), receptor structural alterations impair G-protein coupling efficiency, reduce downstream cAMP signaling (indicated by dashed arrows), and attenuate insulin secretion, resulting in variable clinical outcomes. Central inset: representative distribution of good responders (wild-type) vs poor responders (polymorphic variants) for HbA1c reduction and weight loss. Bottom inset: global geographic distribution of key polymorphism allele frequencies, with Italian populations highlighted. Abbreviations: cAMP, cyclic adenosine monophosphate; GIPR, glucose-dependent insulinotropic polypeptide receptor; GLP1R, glucagon-like peptide-1 receptor; PKA, protein kinase A; SNP, single nucleotide polymorphism.

The functional consequences of GLP1R and GIPR polymorphisms operate through several nonmutually exclusive mechanisms [65]. First, variants affecting receptor expression levels (eg, rs761387, rs761386, and rs2254336) alter the density of pharmacological targets available for drug binding, directly influencing the maximum achievable pharmacodynamic effect [66]. Second, missense variants in the ECD or TMD regions (eg, rs3765467, rs10305492) modify ligand binding affinity or the conformational dynamics of receptor activation, altering the concentration-response relationship [67] (Figure 2).

Third, variants in intracellular domains or those affecting G-protein coupling efficiency (eg, rs6923761, rs1800437) modulate the efficiency of signal transduction downstream of ligand binding [68]. Fourth, variants affecting β-arrestin recruitment kinetics alter the balance between G protein-mediated signaling (mediating insulin secretion and acute glucose lowering) and β-arrestin-mediated signaling (mediating receptor internalization, desensitization, and potentially weight loss through central mechanisms) [44, 69]. This biased signaling concept is particularly relevant for tirzepatide, which exhibits intrinsic GIP receptor bias toward β-arrestin pathways [16].

## Clinical translation and precision medicine

The translation of pharmacogenomic knowledge into clinical practice for incretin therapy personalization faces several challenges [70]. The effect sizes of individual common variants are modest (typically explaining <5% of response variance), necessitating polygenic approaches that aggregate effects across multiple loci [71]. Pharmacogenomic panels incorporating GLP1R and GIPR variants alongside variants in other relevant genes (TCF7L2, KCNJ11, ABCC8, and SLC47A1) show promise for improving response prediction in research settings [72, 73].

For clinical implementation, the most immediately actionable pharmacogenomic finding may be the GIPR rs1800437 (E354Q) association with tirzepatide-induced gastrointestinal adverse effects [42]. Pretreatment genotyping for this variant could identify patients at elevated risk of nausea/vomiting, informing dose escalation strategies and patient counseling [42]. Economic analyses suggest that pharmacogenomic-guided dose optimization could reduce treatment discontinuation rates and improve cost-effectiveness of tirzepatide therapy [74].

The development of clinical pharmacogenomic decision support tools for incretin therapy will require prospective validation studies with adequate statistical power, diverse population representation including Italian and Mediterranean cohorts, and standardized outcome definitions [75, 76]. The Endocrine Society's precision medicine task force has identified pharmacogenomics of GLP-1RAs as a priority research area [77].

## Conclusions and future directions

GLP1R and GIPR polymorphisms represent biologically plausible and clinically relevant determinants of interindividual variability in response to semaglutide and tirzepatide. The evidence base, while growing, remains insufficient for routine clinical pharmacogenomic testing, with most studies limited by modest sample sizes, retrospective designs, and inadequate population diversity [52, 53].

Key priorities for future research include: (1) prospective pharmacogenomic studies in large, ethnically diverse cohorts including Italian and Mediterranean populations; (2) functional characterization of variants identified through GWAS; (3) development and validation of polygenic pharmacogenomic scores for incretin therapy response; (4) investigation of gene–gene and gene–environment interactions; (5) exploration of epigenetic modifications as additional layers of pharmacodynamic variability; and (6) health economic analyses of pharmacogenomic-guided prescribing [44, 52].

The convergence of advanced genomic technologies, large-scale biobanks, and electronic health records creates an unprecedented opportunity to advance precision endocrinology. Realizing the promise of genotype-guided incretin therapy will require coordinated international efforts, with particular attention to ensuring that Italian and Southern European populations are adequately represented in pharmacogenomic discovery and validation cohorts [35].

## Data Availability

No original data were generated or analyzed for this review. All data discussed are from previously published studies, which are cited in the reference list.
